# Age-related gene expression changes, and transcriptome wide association study of physical and cognitive aging traits, in the Lothian Birth Cohort 1936

**DOI:** 10.18632/aging.101333

**Published:** 2017-12-01

**Authors:** Sarah E. Harris, Valentina Riggio, Louise Evenden, Tamara Gilchrist, Sarah McCafferty, Lee Murphy, Nicola Wrobel, Adele M. Taylor, Janie Corley, Alison Pattie, Simon R. Cox, Carmen Martin-Ruiz, James Prendergast, John M. Starr, Riccardo E. Marioni, Ian J. Deary

**Affiliations:** ^1^ Centre for Cognitive Ageing and Cognitive Epidemiology, University of Edinburgh, Edinburgh EH8 9JZ, UK; ^2^ Medical Genetics Section, University of Edinburgh Centre for Genomic and Experimental Medicine and MRC Institute of Genetics and Molecular Medicine, Western General Hospital, Edinburgh EH4 2XU, UK; ^3^ The Roslin Institute and R(D)SVS, University of Edinburgh, Easter Bush Campus, Midlothian EH25 9RG, UK; ^4^ Edinburgh Clinical Research Facility, University of Edinburgh, Western General Hospital, Edinburgh, EH4 2XU, UK; ^5^ Department of Psychology, University of Edinburgh, Edinburgh EH8 9JZ, UK; ^6^ Institute for Ageing, Newcastle University, Campus for Ageing and Vitality, Newcastle upon Tyne NE4 5PL, UK; ^7^ Alzheimer Scotland Dementia Research Centre, University of Edinburgh, Edinburgh EH8 9JZ, UK; ^8^ Queensland Brain Institute, The University of Queensland, Brisbane 4072, QLD, Australia

**Keywords:** cognitive, physical, gene expression, methylation, telomeres, smoking

## Abstract

Gene expression is influenced by both genetic variants and the environment. As individuals age, changes in gene expression may be associated with decline in physical and cognitive abilities. We measured transcriptome-wide expression levels in lymphoblastoid cell lines derived from members of the Lothian Birth Cohort 1936 at mean ages 70 and 76 years. Changes in gene expression levels were identified for 1,741 transcripts in 434 individuals. Gene Ontology enrichment analysis indicated an enrichment of biological processes involved in the immune system. Transcriptome-wide association analysis was performed for eleven cognitive, fitness, and biomedical aging-related traits at age 70 years (N=665 to 781) and with mortality. Transcripts for genes (*F2RL3, EMILIN1* and *CDC42BPA*) previously identified as being differentially methylated or expressed in smoking or smoking-related cancers were overexpressed in smokers compared to non-smokers and the expression of transcripts for genes (*HERPUD1, GAB2, FAM167A* and *GLS*) previously associated with stress response, autoimmune disease and cancer were associated with telomere length. No associations between expression levels and other traits, or mortality were identified.

## INTRODUCTION

This study investigates how gene expression levels 1) change within older age and 2) are associated with age-related physical, cognitive, and biochemical traits. Gene expression levels are influenced by both multiple genetic variants and the environment. They influence most human traits and may provide insight into the biological mechanisms responsible for individual differences. The focus of the present study is on age-related differences.

The UK population is rapidly aging. However, little is known about the molecular mechanisms responsible for individual differences in age-related physical and cognitive traits [1]. As individuals age, many physical traits decline. Decreases in lung function, muscle strength, and gait speed are particularly common, and are associated with morbidity and mortality [2-8]. Age-related cognitive decline is one of the most feared aspects of growing older, and it is highly predictive of dementia, morbidity and mortality [9].

Many physical traits that decline with age are heritable. Twin-based heritability of lung function is estimated to be between 39 and 54% in adults [10, 11], muscle strength between 40 and 65% in older adults [12, 13], and gait speed between 15 and 51% in older women [14, 15]. Recent genome-wide association (GWA) studies have identified genomic regions associated with lung function [16, 17] and grip strength (a commonly used measure of muscle strength) [18]. A GWA study of gait speed did not identify any genome-wide significant signals [19].

Twin based heritability studies estimate the heritability of cognitive function to be greater than 50%, increasing from ∼50% in childhood to as high as ∼80% in middle adulthood and older age [20]. SNP based heritability was shown to be ∼30% in individuals aged over 40 years [21, 22], and a study based on 1,900 members of the Lothian Birth Cohorts of 1921 and 1936 and the Aberdeen Birth Cohort of 1936 estimated SNP based heritability of cognitive decline, between the ages of 11 and 64-79 years, to be ∼24% [23]. GWA studies indicate that both cognitive decline and cognitive ability are highly polygenic traits [21-25].

Oxidative stress is hypothesised to contribute to age-related physical and cognitive decline, possibly through increased inflammation [26] resulting in shortening of telomeres [27]. This may be compounded by smoking [28]. Telomere length is reported to decrease with age [29, 30] and to be influenced by smoking [28], social status and psychological stress [31], although its use as a biological marker of cognitive and physical aging is equivocal [30, 32, 33].

As indicated above, many age-related traits are heritable; however, they are also polygenic, with few specific genetic variants significantly contributing to individual differences. Moreover, they are also influenced by environmental and life-style factors. As well as trying to detect these many small and interacting influences *per se*, an attractive option is to measure an entity that is a summary outcome of their combined effects, and which might have both a more-detectable association with aging and be an indicator of the causal pathways involved. To gain further insight into the mechanisms leading to physical and cognitive aging, researchers are starting to investigate associations between aging-related traits and genome-wide gene expression levels; these are influenced by multiple genetic variants, methylation status, the environment, and gene x gene and gene x environment interactions. Therefore, gene expression levels capture the net influences on transcription and are more likely to be associated with age-related and other traits than are individual genetic variants. A meta-analysis of 7,781 individuals aged 20-104 years, from four cohorts, identified 221 differentially expressed genes associated with muscle strength [34]. Differential gene expression has also been identified in smokers versus never smokers in a meta-analysis of 10,233 individuals from six cohorts [35], which may in turn influence aging. To the best of our knowledge, genome-wide expression levels have not been investigated for other aging traits.

In the present study, we measured transcriptome-wide gene expression levels, using the Illumina HumanHT-12v4 Expression BeadChip, in lymphoblastoid cell lines derived from ∼800 members of the Lothian Birth Cohort 1936 (LBC1936) at age ∼70 years and from ∼400 of the same individuals at age ∼76 years. As far as we are aware, this is the first study to investigate changes in gene expression levels within older age. We next investigated the association of gene expression levels with eleven cognitive, fitness, and biomedical aging-related traits at age 70 years, and with mortality.

## RESULTS

Descriptive statistics for a general fluid cognitive factor (gf), childhood-to-older-age IQ change, time taken to walk six metres, grip strength, forced expiratory volume from the lungs in one second (FEV1), C-reactive protein (CRP), fibrinogen, Glycohemoglobin (HbA1c), smoking status, telomere length, and mortality status are shown in Table 1. Individuals for whom expression data were available at age 70 years differed very little from the whole cohort, in all measures, at age 70 years. Individuals for whom expression data were available at ages 70 and 76 years had higher general cognitive function, a faster walk time, greater grip strength, better lung function, lower levels of fibrinogen, were less likely to have acute levels of CRP, and were less likely to smoke at age 70 years than those for whom only age 70 expression data were available.

**Table 1:** Summary descriptive data for LBC1936

	Full sample		Individuals with age 70 expression data		Individuals with age 70 and 76 expression data	
Variable	Mean (SD, range)	N	Mean (SD, range)	N	Mean (SD, range)	N
Age (years)	69.5 (0.8, 67.6-71.3)	1091	69.5 (0.8, 67.6-71.3)	781	69.5 (0.8, 67.6-71.3)	434
gf	0.0 (1.0, −3.5-2.9)	1072	−0.005 (0.1, −3.0-2.9)	767	0.1 (0.1, −3.0-2.9)	429
IQ change	0.006 (0.9, −5.6-4.4)	1016	0.005 (1.0, −5.6-4.4)	726	0.05 (0.9, −4.0-4.4)	404
6m walk time (s)	3.8 (1.1, 1.1-11.0)	1084	3.8 (1.1, 1.1-10.9)	778	3.7 (0.9, 1.1-8.8)	433
Grip strength (Kg)	29.0 (10.2, 2-60)	1066	29.4 (10.2, 4-60)	768	30.2 (10.0, 11-60)	427
FEV1 (L)	2.4 (0.7, 0.5-5.1)	1085	2.4 (0.7, 0.5-5.1)	780	2.5 (0.7, 0.7-4.3)	434
Fibrinogen (g/L)	3.3 (0.6, 1.6-6.2)	1052	3.3 (0.6, 1.6-6.2)	769	3.2 (0.6, 1.6-5.9)	428
HbA1c (% total)	5.9 (0.7, 4.5-11.6)	1060	5.9 (0.7, 4.5-11.6)	776	5.9 (0.7, 4.5-11.6)	431
Telomere length (kb)	4.2 (0.6, 2.7-7.1)	1070	4.2 (0.5, 2.8-7.1)	781	4.2 (0.6, 2.8-7.1)	434
Sex	Male	548 (50%)		400 (51%)		230 (53%)
	Female	543 (50%)		381 (49%)		204 (47%)
CRP	Normal (0.3 mg/L)	535 (51%)		401 (52%)		244 (57%)
	Elevated (4-10 mg/L)	399 (38%)		294 (38%)		154 (36%)
	Acute (>10 mg/L)	120 (11%)		80 (10%)		34 (8%)
Smoking status	Current smoker	125 (12%)		92 (12%)		33 (8%)
	Past smoker	465 (43%)		325 (42%)		182 (42%)
	Never smoker	501 (46%)		364 (47%)		219 (51%)
Mortality status	Alive	868 (80%)		627 (80%)		398 (92%)
	Dead	223 (20%)		154 (20%)		36 (8%)

### Expression change between ages 70 and 76 years

**Figure 1:**
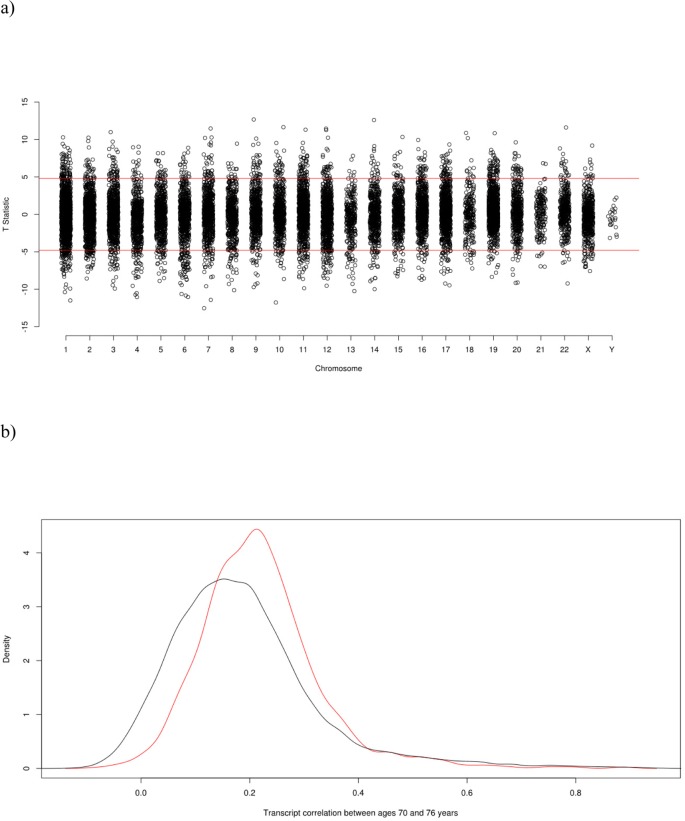
**(a)** Transcriptome-Wide Association Study Manhattan Plot of the paired t-test T-statistic for difference in expression at age 70 and age 76. **(b)** Density Plot of transcript (Pearson) correlations between ages 70 and 76. The red curve is the correlation distribution for the Bonferroni corrected significant transcripts that either increase or decrease in expression levels with age; the black curve is for all transcripts.

**Figure 2:**
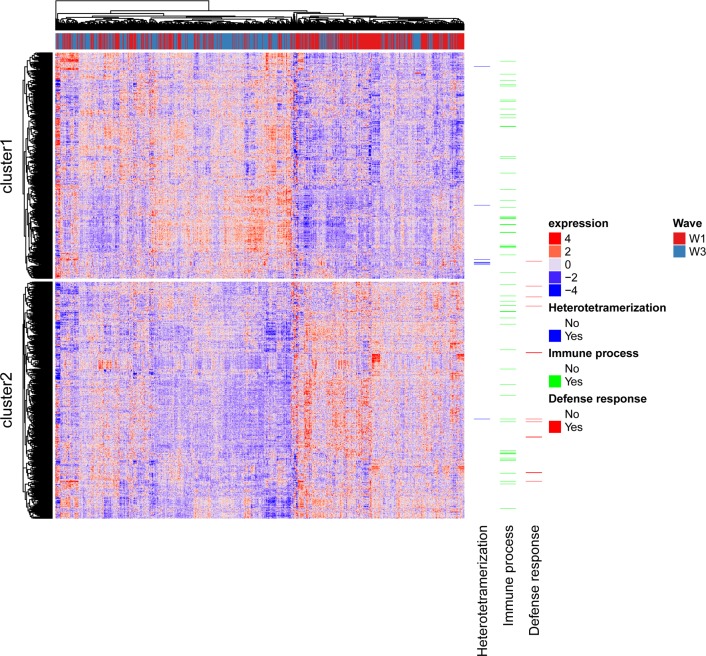
Heatmap of gene expression profiles across individuals. Rows are probes and columns are individuals with the red/blue bar along the top indicating to which wave the column corresponds. Genes linked to the top term identified in each GO analysis are shown.

A significant difference in expression levels between ages 70 and 76 years was identified for 1,741 transcripts, with a negative T value indicating an increase in expression level. 874 showed an increase in expression and 867 a decrease in expression (Figure 1a, Supplementary Table 1). Figure 1b shows a density plot of transcript (Pearson) correlations between ages 70 and 76 years for all transcripts and for Bonferroni corrected significant transcripts only. The correlations are listed in Supplementary Table 1. Correlations for control samples were all > 0.95 within plates and > 0.90 between plates. The expression of the 1,323 probes significantly differentially expressed and successfully mapped to a gene is illustrated in Figure 2, along with the results of the cluster analysis. Cluster analysis separated the genes into those that increase in expression (cluster 1) and those that decrease in expression (cluster 2) between waves 1 and 3. After correction for 13,142 Gene Ontology (GO) terms used in the analysis, GO enrichment analysis using all genes (those showing increased and decreased expression, ranked by p value) indicated enrichment for six biological processes (Supplementary Table 2). The most significant of these were the immune system (p=3.0×10^−9^), defense response (p=1.8×10^−7^), and positive regulation of the immune system (p=5.7×10^−7^) processses. After correction for 10,471 GO terms, enrichment analysis using genes that showed increased expression indicated enrichment for protein heterotetramerization (p=3.4×10^−6^) (Supplementary Table 3). After correction for 11,220 GO terms, enrichment analysis using genes that showed decreased expression indicated enrichment for five biological processes (Supplementary Table 4). The most significant of these were the defense response (p=2.3×10^−7^), inflammatory response (p=7.2×10^−7^), and cytokine-mediated signalling pathway (p=1.1×10^−6^) processes. As expected, there was a bias towards genes linked to heterodimerization in cluster 1 and a bias towards genes linked to defense response in cluster 2 (Figure 2).

### Comparison with a previously published cross-sectional study of genes differentially expressed with age

Of the 1,323 differentially expressed probes mapped to a gene, 1,130 were assigned a unique gene symbol. 907 of these genes were previously reported in a cross-sectional study that identified 1,497 genes differentially expressed with age in the whole blood of 14,983 individuals [36]. 244 of the 907 genes were differentially expressed in this previous study (p<4.2×10^−6^), of which 113 were differentially expressed in the same direction (Supplementary Table 5).

### Transcriptome-wide analyses

**Figure 3:**
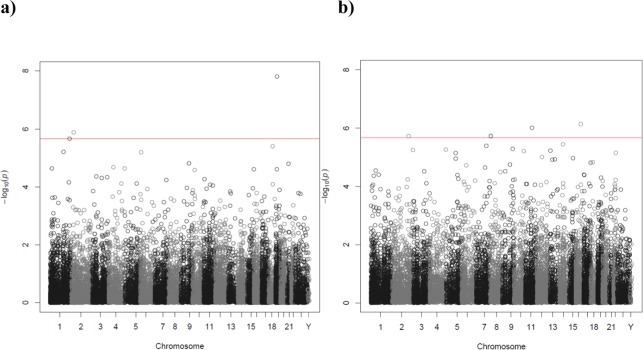
Manhattan plot of the P-values of the transcriptome-wide association analyses for **(a)** smoking status (current versus never) and **(b)** telomere length. The red line indicates the threshold for transcriptome-wide significance (P<2.17×10^−6^).

For the transcriptome-wide association analyses with a sample size of 665 (the smallest N used), we have 80% power (alpha=0.05, two-sided) to detect a correlation of 0.11. Transcriptome-wide association analysis of current smoker versus never smoker identified four transcripts associated with smoking status (Figure 3a, Table 2). Three of the transcripts were annotated to genes: *F2RL3* on chromosome 19 (p=1.57×10^−8^), *EMILIN1* on chromosome 2 (p=1.30×10^−6^) and *CDC42BPA* on chromosome 1 (p=2.16×10^−6^). None of these genes were identified as being differentially expressed in a large (10,233 participants) whole-blood transcriptome meta-analysis of current versus never smokers [35]. Seven of the top 10 genes in the Huan et al. 2016 study [35] were represented by transcripts in this study, of which one (*PYHIN1*, p=0.03) was nominally significantly (p<0.05) associated with smoking status, but in the opposite direction (Supplementary Table 14). No transcripts for the top gene (*LRRN3*) in the Huan et al. 2016 study [35] passed quality control in our study. The full association results are shown in Supplementary Table 14.

**Table 2:** Transcriptome-wide significant genes for current versus never smokers

Probe	Gene symbol	Chr	Beta	St. Err	P
ILMN_2127298	*F2RL3*	19	0.11	0.019	1.57E-08
ILMN_2390946	*NA*	NA	0.076	0.015	2.51E-07
ILMN_1711439	*EMILIN1*	2	0.095	0.019	1.30E-06
ILMN_1781472	*CDC42BPA*	1	0.14	0.029	2.16E-06

No transcripts were associated with former versus never smokers (Supplementary Table 15). Six out of the top 10 genes in the Huan et al. 2016 study [35] were represented by transcripts in this study, of which two (*RASSF1*, p=0.03 and *PILRA*, p=0.02) were nominally significantly (p<0.05) associated with smoking status, but in the opposite direction (Supplementary Table 15).

Transcriptome-wide association identified five transcripts associated with telomere length (Figure 3b, Table 3). The transcripts were for the following genes: *HERPUD1* on chromosome 16 (p=7.32×10^−7^), *GAB2* on chromosome 11 (p=9.81×10^−7^), *FAM167A* on chromosome 8 (p=1.86×10^−6^), *GLS* on chromosome 2 (p=1.92×10^−6^) and *FAM167A* on chromosome 8 (p=1.95×10^−6^). The full association results are shown in Supplementary Table 16.

**Table 3:** Transcriptome-wide significant genes for telomere length

Probe	Gene symbol	Chr	Beta	St. Err	P
ILMN_2374159	*HERPUD1*	16	0.00015	2.98E-05	7.32E-07
ILMN_1665964	*GAB2*	11	7.35E-05	1.49E-05	9.81E-07
ILMN_3248511	*FAM167A*	8	0.00012	2.53E-05	1.86E-06
ILMN_2188722	*GLS*	2	0.00010	2.14E-05	1.92E-06
ILMN_1687213	*FAM167A*	8	0.00011	2.39E-05	1.95E-06

No transcripts were significantly associated with gf, IQ change, six metre walk time, FEV1, grip strength, CRP, fibrinogen, HbA1c, or methylation age acceleration (Hannum or Horvath) (Supplementary Tables 6-13, 17-18). Eight out of the top 10 genes for muscle strength in the Pilling et al. 2016 study of 7,781 participants, [34] were represented by transcripts in this study, of which one (*PNP*, p=0.03) was nominally significantly associated with grip strength at p<0.05, but in the opposite direction (Supplementary Table 10).

### Survival analyses

Gene expression levels were not associated with mortality (all p<2.17×10^−6^) (Supplementary Table 19).

## DISCUSSION

This study has identified 1,741 transcripts that demonstrate a change in expression levels in lymphoblastoid cell lines derived from 434 individuals at ages 70 and 76 years. At age 70 years the expression levels of specific genes were associated with smoking status and telomere length.

The findings from the GO enrichment analyses indicated an enrichment of biological processes involved in the immune system. This is consistent with the finding of a decline in the normal functioning of the immune system with age [37]. A similar number of genes showed increased levels of gene expression as showed decreased levels of gene expression with age, which is in line with previous findings from cross-sectional studies [36, 38], that also identified gene expression changes in genes involved in immune processes. A look-up of the 907 genes that we showed to be differentially expressed with age, and were included in a relatively large (N=14,983) cross-sectional study of expression in whole blood [36], showed that 244 were also differentially expressed in the whole blood study. However, only 113 were differentially expressed in the same direction. Discrepancies in these results are likely due to the different cell types sampled (whole blood which is a mixture of cell types versus LCLs), and the cross-sectional nature of the previous study (mean ages of cohorts included 38-72 years) versus the longitudinal nature of the current study (ages 70-76 years). Our study specifically looked at change over a six year period in the eighth decade of life, whereas the previous study included many younger individuals. The correlation density plot (Figure 1b) of transcript correlations between ages 70 and 76 years indicated a relatively low level of stability that was slightly higher for transcripts for which expression levels significantly changed between these ages than for all transcripts. This may indicate that the transcripts for which change could be detected were more accurately measured. The high correlations between the control samples (> 0.95 within plates and > 0.90 between plates) indicated that overall, measurement error was small.

The expression of three genes was upregulated in current versus never smokers. *F2RL3,* which showed the strongest association with smoking status, encodes a member of the protease-activated receptor subfamily, part of the G-protein coupled receptor 1 family of proteins that is associated with inflammation. Hypomethylation of *F2RL3* has been associated with smoking status and lung cancer [39, 40]. *CDC42BPA* encodes a member of the Serine/Threonine protein kinase family and is upregulated in pancreatic cancer, a smoking related cancer, compared to normal tissue [41]. *EMILIN1* encodes an extracellular matrix glycoprotein associated with elastic fibers. It has been shown to decrease tumor cell growth [42].

None of the transcripts associated with smoking status in our study were identified in a previous whole-blood transcriptome meta-analysis, including more than 10,000 individuals, of current versus never smokers [35], and none of the top 10 hits from that study were nominally significant in our study. This may be due to the previous study investigating gene expression in whole blood, whereas our study investigated gene expression in LCLs. Our failure to replicate previously published associations for both smoking [35] and muscle strength [34] may also be due to the smaller size of our study.

The upregulation of expression of four genes was associated with longer telomere length. *HERPUD1* encodes a protein that is important in endoplasmic reticulum (ER) stress response. ER stress influences number of diseases including neurodegeneration and cardiovascular disease [43]. *GAB2* encodes a member of the GRB2-associated binding protein (GAB) gene family and is associated with human tumorigenesis, particularly in breast cancer, leukemia and melanoma [44]. *FAM167A* is located in a locus previously associated with autoimmune diseases [45]. *GLS* encodes the K-type mitochondrial glutaminase which is regulated by oncogenes and supports tumor cell growth [46]. No genes previously associated with telomere length via genome-wide association studies [47-49] were shown to have expression levels associated with telomere length in this study.

Strengths of this study include the longitudinal nature, which allowed us to investigate expression changes within older age. The large number of aging variables allowed us to investigate the association between gene expression levels and many cognitive and physical traits in older age, the majority of which had not previously been investigated. LBC1936 have a narrow age range and are genetically homogeneous, reducing variability that may be introduced in other cohorts.

Limitations include the use of LCLs rather than whole blood. However, although we did not identify the same genes as a whole-blood transcriptome meta-analysis of smoking status [35], the majority of the genes that we did identify are known to be differentially methylated or expressed in smokers or smoking related cancers, suggesting that the results are not just artefacts introduced by LCL generation. Other limitations include the relatively short time period between the two waves of blood collection (∼6 years), and the relatively small number of individuals for whom we had longitudinal data (n=434). The number of individuals in each of the transcriptome-wide associations of aging traits ranged from 665 to 781, which means that we had limited power to detect individual small associations, which, when combined, might predict aging-related traits. A further limitation is the relative good health of the LBC1936 participants, thus reducing the variance of these traits relative to the general population. In the future, sample sizes for this type of transcriptomic analysis will increase as other studies with well characterized aging cohorts move onto wide-range expression analysis.

In conclusion, we identified several thousand genes which show either increased or decreased expression between ages of 70 and 76 years. These genes were enriched for immune system processes. In the future we intend to examine associations between changes in expression and changes in cognitive and physical traits. We also showed that genes previously identified as being differentially methylated or expressed in smoking or smoking-related cancers are over expressed in smokers compared to non-smokers in LBC1936. We identified a number of genes with expression levels associated with telomere length that have previously been associated with stress response, autoimmune disease, and cancer. Finally, we have made available results from the first transcriptome-wide associations of a number of age-related physical and cognitive traits that may be used in future meta-analyses of these traits.

## MATERIALS AND METHODS

### Lothian Birth Cohort 1936 (LBC1936) Ethics statement

Investigation has been conducted in accordance with the ethical standards and according to the Declaration of Helsinki and according to national and international guidelines and has been approved by the authors' institutional review board. Ethics permission was obtained from the Multi-Centre Research Ethics Committee for Scotland (Wave 1: MREC/01/0/56), the Lothian Research Ethics Committee (Wave 1: LREC/2003/2/29), and the Scotland A Research Ethics Committee (Wave 3: 07/MRE00/58). All persons gave their informed consent prior to their inclusion in the study.

### Subjects

LBC1936 consists of 1091 (543 female) individuals, most of whom took part in the Scottish Mental Survey of 1947, during which, at the age of ∼11years, they took the Moray House Test version 12 (MHT), a validated test of cognitive ability [50]. At a mean age of 69.5 years (SD 0.8) they were recruited to a study to determine influences on cognitive aging [51, 52]. They underwent a series of cognitive and physical tests. Three further waves of cognitive and physical testing have occurred at mean ages 73, 76, and 79 years. For this study Peripheral Blood Mononuclear Cells (PBMCs) were extracted from whole blood at ages 70 (Wave 1) and 76 (Wave 3) for the generation of lymphoblastoid cell lines (LCLs) at the Edinburgh Clinical Research Facility (ECRF) Genetics Core, Western General Hospital, Edinburgh. DNA extracted from whole blood at age 70 was used to measure telomere length and genome-wide methylation.

### Physical tests

Physical trait measures included time taken to walk six metres as quickly as possible, grip strength measured with a Jamar Hydraulic Hand Dynamometer [all subject had three trials with both hands, and the best of the three trials in the dominant hand (self-reported) was used], and FEV1 measured using a microspirometer (the best of the three trials was used).

### Cognitive tests

Cognitive tests included six Wechsler Adult Intelligence Scale-IIIUK (WAIS-III) [53] non-verbal subtests (matrix reasoning, letter number sequencing, block design, symbol search, digit symbol, and digit span backward). From these six cognitive tests a general fluid cognitive factor (gf) was derived. The scores from the first unrotated component of a principal components analysis were extracted and labelled as gf. This component explained 52% of the variance, with individual test loadings ranging between 0.65 and 0.72. MHT scores from ages 11 and 70 were corrected for age in days and converted to MHT IQ-type scores (mean = 100; SD = 15). IQ change between ages 11 and 70 years was then calculated by regressing age 11 IQ on age 70 IQ.

### Biochemical tests

CRP was measured using a dry slide immunorate method on the OrthoFusion 5.1 F.S. analyser (Vitros Chemistry Products CRP slides, Ortho Clinical Diagnostics, Buckinghamshire, UK). As the CRP assay is designed for detecting raised levels of CRP it cannot distinguish values less than 3mg/L and all readings less than 3mg/L (N = 467) were assigned a value of 1.5 mg/L. The reference interval for the assay is 10mg/L. Therefore, CRP levels were classified as: normal (0-3mg/L), elevated (4-10mg/L) or acute (>10mg/L) as previously published [54]. Fibrinogen was measured using an automated Clauss assay (TOPS coagulator, Instrumentation Laboratory, Warrington, UK). HbA1c was measured in non-fasting blood using an Adams HA-8160 HbA1c analyzer, utilizing HPLc. The coefficients of variation for these biomarkers are: CRP = 126%, fibrinogen = 20%, HbA1c = 13%. Smoking status was recorded as current smoker, former smoker or never smoker.

Extreme outliers were removed after visual inspection of the data. Two outliers were removed from HbA1c and one each from six metre walk time and IQ change.

### Isolation of peripheral blood mononuclear cells

Whole blood was collected in 9ml Lithium Heparin tubes with PBMC isolation occurring within 72 hours of venepuncture. Whole blood was layered on histopaque and span at 400g for 30 mins at room temperature. Separated cells were collected and washed in RPMI (1% 100x Penicillin/Streptomycin and 1% L-Glutamine) and re-suspended in 1ml freezing mix (8% DMSO in Fetal Calf Serum). Samples were slowly frozen in a ULT using a cryo freezing container for at least 4 hours before transferring to Liquid Nitrogen storage. Cell number and viability was assessed using a haemocytometer and trypan blue exclusion viability test.

### Generation of lymphoblastoid cell lines

PBMCs underwent Epstein Barr Transformation of the B-lymphocyte component to generate LCLs at the European Collection of Cell Cultures, Public Health England, Porton Down, using standard methods [55]. Frozen cell pellets were returned to the ECRF Genetics Core for RNA extraction.

### RNA extraction

RNA was extracted from LCL pellets using Qiagen miRNeasy kit. Cells were homogenised in Qiazol Buffer with chloroform added to the homogenate and phase separation performed at 4°C. The upper aqueous phase was added to 1.5x ethanol and mixed, before being added to the column. DNase treatment was on-column. Eluted RNA was stored in a ULT. RNA yield and RIN were assessed by Agilent 2100 Bioanalyser and Nanodrop.

### Gene expression profiling

Samples were amplified and biotin labelled using the Ambion Illumina Totalprep RNA Amplification Kit. The quality of the labelled cDNA was assessed on the Agilent Bioanalyser and genome-wide gene expression levels were measured on good quality samples using the Illumina HumanHT-12 v4 Expression BeadChip and scanned on an Illumina HiScan running GenomeStudio v2011.1. A control RNA sample was included on each array. The following quality control (QC) procedures were applied to transcript profiles. Individuals with signal-to-noise ratio <10 or with <9,000 transcripts (P<0.01) detected were excluded (n=130). Probes present in >20% of individuals with P<0.05 were retained. The lumiExpresso command in the R package ‘lumi’ [56] was used to perform variance stabilising transformation and quantile normalisation within a single step. After QC 23,031 transcripts were retained in 781 individuals at age 70 years and 574 individuals at age 76 years. Transcript sequences were aligned to the build 38 reference genome using BLAT [57]. Only probes that perfectly mapped to a unique location in the genome were kept, with all probes mapping to a second position with greater than 40% identity excluded. The remaining transcripts were annotated to genes by overlapping their locations with Ensembl build 88 gene coordinates using BEDtools v2.26.0 [58]. 14,500 transcripts that passed QC were successfully annotated to a gene.

### Telomere length measurement

Telomere length was measured using a quantitative real-time polymerase chain reaction (PCR) assay [59]. The intra-assay coefficient of variation was 2.7% and the inter-assay coefficient of variation was 5.1%. Four internal control DNA samples were run within each plate to generate absolute telomere lengths and to correct for plate to plate variation. These internal controls are cell lines of known absolute telomere length, 6.9kb, 4.03kb, 2.0kb and 1.32kb respectively, whose relative ratio values (telomere starting quantity/glyceraldehyde 3-phosphate dehydrogenase starting quantity) were used to generate a regression line by which values of relative telomere length for the actual samples were converted into absolute telomere lengths. The correlation between relative telomere length and absolute telomere length was 0.8. Measurements were performed in quadruplicate and the mean of the measurements used. PCRs were performed on an Applied Biosystems (Pleasonton, CA, USA) 7900HT Fast Real Time PCR machine.

### Methylation

Measurement of DNA methylation in LBC1936 has been described in detail previously [60]. Briefly genome-wide methylation levels were measured in DNA extracted from whole blood using the Illumina HumanMethylation450 BeadChips array. Quality control was carried out on these data to remove (i) probes with a low detection rate, (ii) low quality samples, (iii) samples with a low call rate, (iv) samples where there was a sex mismatch. Post-QC there were 450,726 autosomal probes available for analysis in 920 participants. Seventy-one of these probes were used to calculate DNA methylation age using the regression weights supplied by Hannum et al. [61]. 353 of these probes were used to calculate DNA methylation age using the regression weights supplied by Horvath et al [62]. Methylation age acceleration for both the Hannum and the Horvath methylation ages was calculated by regressing DNA methylation age on chronological age and saving the residual.

### Statistical analyses

#### Expression change between ages 70 and 76 years

A paired t-test was used to investigate the difference in the expression of each transcript between ages 70 and 76 years for the 434 individuals with expression measured at both ages. Adjustment was made for plate and batch. A Bonferroni corrected p value of 0.05/n_transcripts_ = 0.05/23031 =2.17×10^−6^ was used to indicate statistical significance. All genes were entered into a Gene Ontology (GO) enrichment analysis, ranked by p value of the transcript with the lowest p value, for biological processes using the Gene Ontology enRIchment anaLysis and visuaLizAtion tool (GORILLA) [63, 64]. Next, separate analyses were performed for genes where expression levels increased and for genes where expression levels decreased. No p value cut off was used to select genes that were entered into the GO enrichment analyses. A Pearson correlation was calculated for each transcript at age 70 and 76 years, controlling for plate and batch.

A heatmap of gene expression profiles across individuals and waves was plotted using the ComplexHeatmap package in R [65]. Plotting was restricted to genes significantly differentially expressed between waves across the 434 individuals with expression data at both timepoints. Both rows and columns of the heatmap were scaled to a mean of 0 and standard deviation of 1 prior to plotting. K means clustering with k set to two was used to separate genes into those going up and down between timepoints and GO terms were retrieved using the biomaRt R package [66].

#### Transcriptome-wide analyses

Power calculations were performed using the pwr R package [67, 68]. Linear regression models were used to test the association of gene expression at age 70 years with gf, IQ change between the ages of 11 and 70 years, six metre walk time, FEV1, grip strength, CRP, fibrinogen, HbA1c, smoking status (current versus never and former versus never), telomere length and methylation age acceleration (Hannum and Horvath), adjusting for age, sex, batch and plate (N=665 to 781). Grip strength, six metre walk time and FEV1 were also adjusted for height. Expression of each gene was the outcome variable in all models. A Bonferroni corrected p value of 2.17×10^−6^ was used to indicate statistical significance. Linear regression analyses were performed in R. The most significant transcripts for the top 10 genes associated with muscle strength in [34] and smoking status (current versus never and former versus never) in [35] were identified in our equivalent analyses.

#### Survival analyses

Mortality status was obtained from data linkage from the National Health Service Central Register, provided by the General Register Office for Scotland (now National Records of Scotland). Cox proportional hazards regression models were used to test the association between gene expression and mortality, adjusting for age at sample collection, sex, batch, and plate. Survival analyses were performed in R [68] using the survival package [69].

## SUPPLEMENTARY MATERIAL TABLES


